# Primary hepatic alpha-fetoprotein-producing neuroendocrine neoplasm harboring *FGFR2* and *TP53* mutations: a case report and literature review

**DOI:** 10.1007/s00795-024-00408-w

**Published:** 2024-11-10

**Authors:** Hirofumi Watanabe, Kodai Enda, Fumiyoshi Fujishima, Hidekazu Shirota, Masashi Ninomiya, Tetsuro Yamazaki, Hironobu Sasano, Takashi Suzuki

**Affiliations:** 1 Department of Pathology, Tohoku University Hospital, Sendai, Miyagi Japan; 2 Department of Medical Oncology, Tohoku University Hospital, Sendai, Miyagi Japan; 3 Division of Gastroenterology, Tohoku University Graduate School of Medicine, Sendai, Miyagi Japan; 4 Department of Diagnostic Radiology, Tohoku University Hospital, Sendai, Miyagi Japan; 5 Department of Anatomic Pathology, Tohoku University Graduate School of Medicine, Sendai, Miyagi Japan

**Keywords:** Hepatic neuroendocrine neoplasm, AFP, FGFR2, TP53

## Abstract

This report presents a rare case of a 45-year-old man diagnosed with a primary hepatic alpha-fetoprotein-producing neuroendocrine neoplasm, a condition rarely reported in the literature. The patient presented with initial symptoms of back and epigastric pain, after which multiple liver lesions were discovered on contrast-enhanced computed tomography, suggesting intrahepatic cholangiocarcinoma. Histopathological and immunohistochemical analyses confirmed the diagnosis of alpha-fetoprotein-producing neuroendocrine neoplasm that was further supported by genetic testing, which revealed *FGFR2* and *TP53* mutations commonly encountered in intrahepatic cholangiocarcinoma. Despite receiving various chemotherapeutic regimens, the patient exhibited a progressive disease. This case underscores the importance of accurate differential diagnosis from hepatocellular carcinoma and intrahepatic cholangiocarcinoma due to differences in treatment approaches and prognoses and highlights the necessity for increased awareness of AFP-producing primary hepatic neuroendocrine neoplasms among clinicians and pathologists. It emphasizes the significance of comprehensive histopathological evaluation, immunohistochemical profiling, and genetic analysis for precise diagnosis and tailored therapeutic strategies. Further research is warranted to elucidate the molecular mechanisms underlying this rare liver tumor subtype and develop targeted treatments.

## Introduction

### Case presentation

**Fig. 1:**
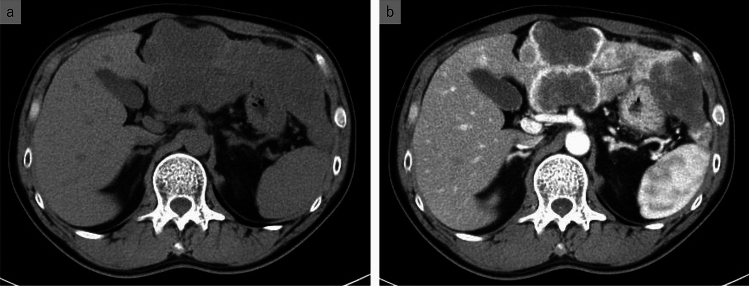
Radiologic findings of a primary hepatic alpha-fetoprotein-producing neuroendocrine neoplasm. **a** Pre-contrast CT image reveals multiple tumorous lesions within the liver. **b** Contrast-enhanced arterial phase of the CT scan. Strong contrast enhancement is evident at the periphery of the occupying lesion, while the interior shows hypovascularity. In addition, the tumor periphery exposed to the liver surface exhibits a concave indentation. These findings are consistent with those of intrahepatic cholangiocarcinoma. *CT* computed tomography

A 45-year-old man presented with intermittent back and epigastric pain. Upper gastrointestinal endoscopy revealed gastritis, the symptoms of which persisted post-treatment. Subsequently, contrast-enhanced computed tomography (CT) revealed multiple lesions in the liver (Fig. 1a, b). The largest tumor (nearly 12 cm in diameter) was located in the lateral segment of the left lobe and was accompanied by multiple additional tumors measuring 1–6 cm. Dynamic contrast-enhanced imaging showed intense early-phase enhancement along the periphery of the occupying lesions. However, the internal areas appeared to be hypovascular. Moreover, the tumor periphery exposed to the liver surface exhibited a concave indentation, consistent with intrahepatic cholangiocarcinoma. The largest tumor was assumed to be the primary site, and the others were deemed metastases. The differential diagnosis included multiple liver metastases from malignancies originating outside the liver; however, no extrahepatic lesions suggestive of a primary tumor were identified. The clinical findings at admission are summarized in Table 1. Blood tests revealed elevated levels of alpha-fetoprotein (AFP; 7683.0 ng/mL) and neuron-specific enolase (21.4 ng/mL).

**Table 1:** Laboratory findings

WBC 8500 μL	UN 16 mg/dL	Free T4 1.20 ng/dL
Hb 15.9 g/dL	Cre 0.88 mg/dL	hTSH 5.200 μIU/mL
RBC 5320000 μL	UA 5.0 mg/dL	Free T3 3.12 pg/mL
PLT 307000 μL	TP 7.3 g/dL	S HBs-Ab (−)
T-BIL 0.4 mg/dL	ALB 4.8 g/dL	S HBs-Ag (−)
ALP 189U/L	Na 140 mmol/L	HCV-Ab 0.1
D-BIL < 0.1 mg/dL	K 5.0 mmol/L	NSE 21.4 ng/mL
G-GTP 185 U/L	CL 99 mmol/L	AFP 7683.0 ng/mL
AST 57 U/L	HbA1cNGSP 6.1%	CEA 1.9 ng/mL
ALT 62 U/L	T-Cho 161 mg/dL	CA19-9 6.1 U/mL
LDH 249 U/L	TG 88 mg/dL	PIVKA-II 51 mAU/mL
ChE 345 U/L	HDL-C 55 mg/dL	Pro GRP 46.8 pg/mL
Lipase 28 U/L		
Amylase 82 U/L		

*WBC* white blood cell, *Hb* hemoglobin, *RBC* red blood cell, *PLT* platelet, *T-BIL* total bilirubin, *ALP* alkaline phosphatase, *D-BIL* direct bilirubin, *G-GTP* gamma-glutamyl transpeptidase, *AST* aspartate aminotransferase, *ALT* alanine aminotransferase, *LDH* lactate dehydrogenase, *ChE* cholinesterase, *UN* urea nitrogen, *Cre* creatinine, *UA* uric acid, *TP* total protein, *ALB* albumin, *Na* sodium, *K* potassium, *CL* chloride, *HbA1c* hemoglobin A1c, *T-Cho* total cholesterol, *TG* triglyceride, *HDL-C* high-density lipoprotein cholesterol, *Free T4* free thyroxine, *hTSH* human thyroid-stimulating hormone, *Free T3* free triiodothyronine, *S HBs-Ab* surface hepatitis B antibody, *S HBs-Ag* surface hepatitis B antigen, *HCV-Ab* hepatitis C virus antibody, *NSE* neuron-specific enolase, *AFP* alpha-fetoprotein, *CEA* carcinoembryonic antigen, *CA19-9* cancer antigen 19-9, *PIVKA-II* protein induced by vitamin K absence or antagonist-II, *Pro GRP* Pro-gastrin-releasing peptide

**Fig. 2:**
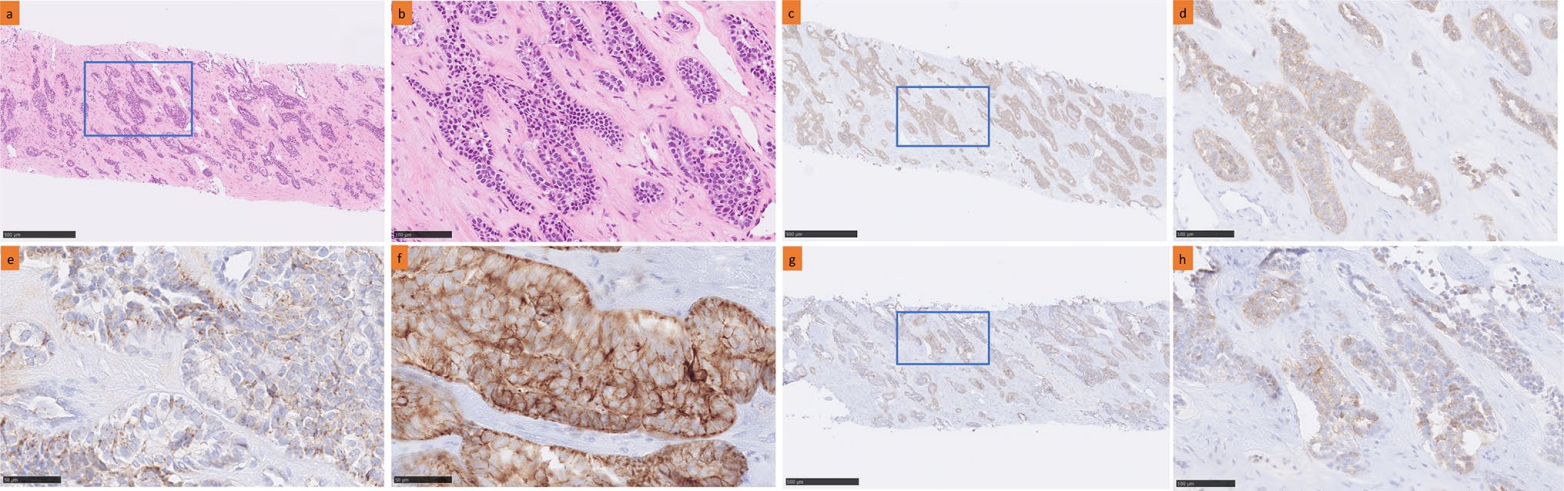
Histopathology of primary hepatic alpha-fetoprotein-producing neuroendocrine neoplasm. **a** Low-magnification images of the liver biopsy (scale bar = 500 μm). **b** High-power view of the selected area (*blue square*) in **a**. Histologic findings reveal atypical cells with abundant cytoplasm and enlarged nuclei forming cord-like structures or a nested organoid growth pattern (scale bar = 100 μm). Immunohistochemically, the tumor stained positive for **c**, **d** synaptophysin, **e** chromogranin A, **f** CK19, and **g**, **h** alpha-fetoprotein (**c**, **g**: scale bar = 500 μm, **d**, **h**: scale bar = 100 μm, **e**, **f**: scale bar = 50 μm). **d** High-power view of the selected area (*blue square*) in **c**. **h** High-power view of the selected area (*blue square*) in **g**. **c** Almost all neoplastic cells stained positive for synaptophysin. **g** About half of the neoplastic cells stained positive for alpha-fetoprotein

Histopathological examination of the liver biopsy specimen showed proliferating atypical cells with abundant cytoplasm and enlarged nuclei, forming cord-like structures or a nested organoid growth pattern with surrounding fibrosis (Fig. 2a, b). Tumor necrosis or degeneration was not apparent (Fig. 2a). Immunohistochemical analysis revealed cells positive for neuroendocrine markers, including synaptophysin (Fig. 2c, d), chromogranin A (Fig. 2e), CK19 (Fig. 2f), and AFP (Fig. 2g, h). Most neoplastic cells were positive for synaptophysin (Fig. 2c, d), and about half of the neoplastic cells were positive for AFP (Fig. 2g). The Ki-67 labeling index was 25% (hot spot). Therefore, the patient was diagnosed with an AFP-producing neuroendocrine neoplasm. Foundation One Liquid CDx test results were as follows: microsatellite instability-high, not detected; tumor mutational burden, intermediate (7.59 Muts/Mb); *FGFR2* missense variant, c.1144T>C; *PTEN* missense variant, c.335T>C; and *TP53* missense variant, c.722C>T. *FGFR2* and *TP53* mutations are common in intrahepatic cholangiocarcinomas. The coexistence of these genetic abnormalities with radiographic features resembling intrahepatic cholangiocarcinoma implied the possibility of mixed neuroendocrine neoplasm (MINEN) with concomitant intrahepatic cholangiocarcinoma.

## Discussion

This report describes a rare case of a patient diagnosed with primary hepatic AFP-producing neuroendocrine neoplasm. Prior to this study, only one case of an AFP-producing neuroendocrine neoplasm originating from the liver has been reported in the literature [1] (Table 1). Primary hepatic neuroendocrine neoplasms are rare and present diagnostic challenges; hence, accurate diagnostic differentiation from other primary malignant liver tumors, such as hepatocellular carcinoma and intrahepatic cholangiocarcinoma, is crucial. The neoplasm in this study showed neuroendocrine differentiation on immunohistochemistry and *FGFR2* and *TP53* mutations on genetic analysis. The mechanisms underlying the acquisition of AFP production in neuroendocrine neoplasms remain unclear; however, the identification of genetic abnormalities commonly associated with intrahepatic cholangiocarcinoma suggests a potential derivation from AFP-producing intrahepatic cholangiocarcinoma [2]. *TP53* mutations are suggestive of neuroendocrine carcinoma rather than neuroendocrine tumor [3]. Therefore, this neoplasm may be a partial MINEN lesion with concomitant intrahepatic cholangiocarcinoma. However, despite the extremely high serum AFP levels, the AFP expression on tumor cells appeared relatively weak (Fig. 2g, h). A limitation of this study is the inability to exclude the possibility of hepatocellular carcinoma at sites other than the biopsy site. In addition, AFP may serve as a prognostic factor for neuroendocrine tumors [4–6]. In this case, chemotherapeutic regimens comprising gemcitabine/cisplatin, S-1 (tegafur, gimeracil, and oteracil potassium), and pemigatinib were administered; however, all yielded a treatment response categorized as progressive disease (Table 2).

**Table 2:** A list of cases of AFP-producing hepatic neuroendocrine neoplasm reported in the literature

Author, year	Case no	Age	Sex	Chief complaint	Size	Ki-67
Huang et al., 2020 [1]	1	42	Male	Persistent upper abdominal pain	14 cm	80%
Present case	2	45	Male	Intermittent back and epigastric pain	12 cm	25%

*AFP* alpha-fetoprotein

AFP-producing hepatic neoplasms warrant extra caution, and this case emphasizes the importance of considering primary hepatic neuroendocrine neoplasms in the differential diagnosis of liver tumors. The presence of AFP production and genetic abnormalities reiterates the importance of accurate differentiation from hepatocellular carcinoma and intrahepatic cholangiocarcinoma, as their treatment approaches and prognoses differ. Moreover, detailed histopathological evaluation, immunohistochemical profiling, and genetic analysis are crucial for accurate diagnosis. Further studies are warranted to elucidate the underlying molecular mechanisms and to develop targeted therapeutic strategies for this subtype of primary hepatic neuroendocrine neoplasm. Increased awareness among clinicians and pathologists can ensure early recognition and appropriate tumor management.

## Data Availability

Data sharing is not applicable to this article, as no datasets were generated or analyzed during the current study.
